# Are Methanol-Derived Foliar Methyl Acetate Emissions a Tracer of Acetate-Mediated Drought Survival in Plants?

**DOI:** 10.3390/plants10020411

**Published:** 2021-02-23

**Authors:** Rebecca A. Dewhirst, Joseph Lei, Cassandra A. Afseth, Cristina Castanha, Christina M. Wistrom, Jenny C. Mortimer, Kolby J. Jardine

**Affiliations:** 1 Climate and Ecosystem Sciences Division, Lawrence Berkeley National Laboratory, Berkeley, CA 94720, USA; josephlei@lbl.gov (J.L.); cafseth2@illinois.edu (C.A.A.); ccastanha@lbl.gov (C.C.); kjjardine@lbl.gov (K.J.J.); 2 School of Integrative Biology, University of Illinois at Urbana-Champaign, Champaign, IL 61801, USA; 3 College of Natural Resources, University of California, Berkeley, CA 94704, USA; cwistrom@berkeley.edu; 4 Environmental Genomics and Systems Biology, Biosciences Division, Lawrence Berkeley National Laboratory, Berkeley, CA 94720, USA; jcmortimer@lbl.gov; 5 School of Agriculture, Food and Wine, Waite Research Institute, University of Adelaide, Glen Osmond, SA 5005, Australia

**Keywords:** biogenic, volatile organic compound, acetylation, methanol, acetate fermentation

## Abstract

Upregulation of acetate fermentation in plants has recently been described as an evolutionarily conserved drought survival strategy, with the amount of acetate produced directly correlating to survival. However, destructive measurements are required to evaluate acetate-linked drought responses, limiting the temporal and spatial scales that can be studied. Here, ^13^C-labeling studies with poplar (*Populus trichocarpa*) branches confirmed that methyl acetate is produced in plants from the acetate-linked acetylation of methanol. Methyl acetate emissions from detached leaves were strongly stimulated during desiccation, with total emissions decreasing with the leaf developmental stage. In addition, diurnal methyl acetate emissions from whole physiologically active poplar branches increased as a function of temperature, and light-dark transitions resulted in significant emission bursts lasting several hours. During experimental drought treatments of potted poplar saplings, light-dark methyl acetate emission bursts were eliminated while strong enhancements in methyl acetate emissions lasting > 6 days were observed with their initiation coinciding with the suppression of transpiration and photosynthesis. The results suggest that methyl acetate emissions represent a novel non-invasive tracer of acetate-mediated temperature and drought survival response in plants. The findings may have important implications for the future understanding of acetate-mediated drought responses to transcription, cellular metabolism, and hormone signaling, as well as its associated changes in carbon cycling and water use from individual plants to whole ecosystems.

## 1. Introduction

Acetylation of metabolites and biopolymers dramatically alters their physical properties such as volatility and solubility, chemical reactivities, and numerous biological properties ranging from the protein function to signaling during abiotic and biotic stress [1]. For example, the regulation of protein acetylation is crucial for many important cellular processes [2]. In addition to biopolymers, lower molecular weight compounds such as alcohols can also be acetylated including the volatile acetate ester (Z)-3-hexenyl acetate, which triggers the expression of defense-related genes [3] and can be produced de-novo upon exposure to wound-induced volatiles [4]. 

A large diversity of volatile acetate esters are produced in plants including short and medium chain acetate esters [5,6], monoterpene acetate esters [7], benzoid esters [8], and acetylated green leaf volatiles [9]. Volatile acetate esters are often described as having pleasant fragrances and are therefore key compounds influencing flower and fruit aroma [10,11,12]. While the majority of studies on plant volatile acetate esters have focused on their content in fruits, flowers, leaves, and essential oils, studies quantifying emissions of acetate esters into the atmosphere are less common. Thus, their identities, emission rates, and biological and environmental drivers from managed and natural ecosystems remain poorly characterized. Terrestrial fluxes of carbon dioxide (CO_2_) and volatile organic compounds (VOCs) between the biosphere and atmosphere are dramatically changing in response to climate factors such as trends in surface warming and a higher frequency and intensity of large-scale droughts [13]. Given that plant mortality risk increases when droughts co-occur with high temperature anomalies [14], the role of volatile acetate esters in drought and high temperature tolerance is noteworthy due to their ability to diffuse rapidly within and between cells, tissues, and the atmosphere. 

There is mounting evidence for a central role of acetate-linked metabolism during plant drought response. In *Arabidopsis thaliana*, a dynamic metabolic switch from glycolysis to acetate fermentation was shown to confer drought tolerance by coordinating whole-plant reprogramming of transcription, cellular metabolism, hormone signaling, and chromatin modification [15]. The amount of acetate produced by plants during drought was found to directly correlate to survivability. Moreover, transgenic plants with mutations in two key genes involved in the acetate fermentation pathway, *pyruvate decarboxylase1* (*pdc1*) and *aldehyde dehydrogenase 2b7* (*aldh2b7*), did not show a significant drought-induced increase in acetate levels and were more sensitive to drought relative to wild-type plants [16]. The exogenous acetic acid application to *A. thaliana*, rapeseed (*Brassica napus*), maize (*Zea mays*), rice (*Oryza sativa*), and wheat (*Triticum aestivum*) plants confirmed a mechanism where acetic acid promotes *de novo* jasmonic acid (JA) synthesis and primes the JA signaling pathway for drought tolerance via the enrichment of histone H4 acetylation [15]. The authors concluded that this novel acetate function is evolutionarily conserved as a survival strategy against drought in plants [15]. Further, the engineered increased expression of the acetic acid pathway genes pyruvate decarboxylase (PDC) and aldehyde dehydrogenase (ALDH) in *Arabidopsis* resulted in improved survival under drought stress [16]. The physiological impacts of increased acetate availability during drought were further investigated in soybean where foliar acetic acid sprays (20 mM) promoted drought acclimation by reducing oxidative stress while enhancing root biomass, leaf area, photosynthesis rates, and water use efficiency leading to improved growth performance [17].

Acetyl CoA (acetyl coenzyme A) is the major acetyl donor in plants and is found in the cytosol and organelles such as the mitochondria and plastids [18]. Methyl acetate is the simplest of the volatile acetate esters and has been previously hypothesized to derive from the acetylation of methanol by acetyl-CoA [1]. Methanol, the simplest alcohol and natural product from plants, is a highly abundant C_1_ metabolite widely considered to derive from pectin demethylation reactions during cell wall physicochemical/structural changes [19]. Although numerous reports of methanol emissions from vegetation exist, methyl acetate has rarely been reported as an emission from foliage, although branch emissions up to 0.5 nmol m^−2^ s^−1^ during the daytime were observed from the Mediterranean shrub *Halimium halimifolium* [1]. Consistent with the view that the acetyl moiety derives from the decarboxylation of pyruvate during acetate fermentation, strong ^13^C-labelling of methyl acetate emissions from *H. halimifolium* occurred only under [2-^13^C]pyruvate- and [2,3-^13^C]pyruvate branch feeding, but not [1-^13^C]pyruvate feeding [1]. Confirmation that the acetyl-moiety can derive from acetate/acetyl-CoA was recently obtained from branch feeding of the tropical pioneer species *Inga edulis* with [1,2-^13^C]acetate that stimulated strong foliar emissions of [^13^C_2_]methyl acetate that greatly exceeded unlabeled methyl acetate emissions [20]. In addition, consistent with methanol as the source of the methoxy moiety of methyl acetate, labeling with [^13^C]methanol resulted in strong foliar emissions of [^13^C_1_]methyl acetate that exceeded unlabeled methyl acetate emissions. 

**Figure 1:**
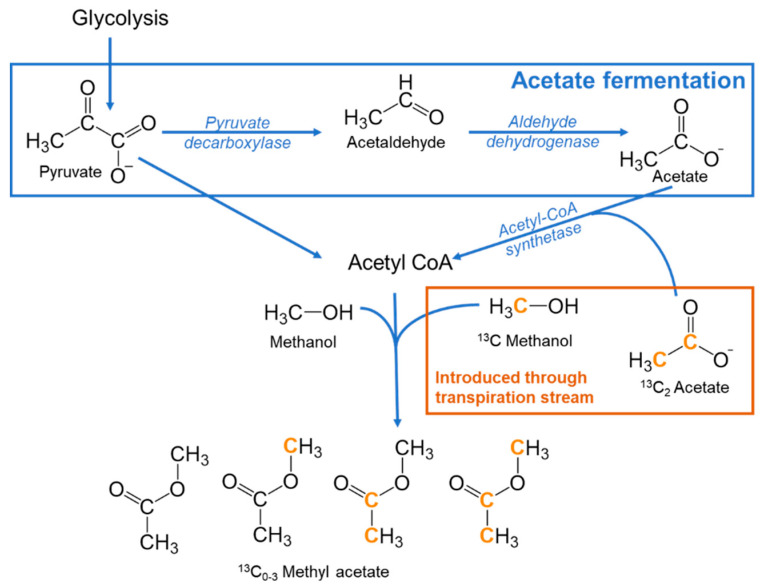
Schematic of acetate fermentation pathway and methyl acetate production. The addition of ^13^C methanol and ^13^C_2_ acetate during these experiments are indicated in the orange box. Labeled ^13^C atoms of the exogenous ^13^C methanol and acetate are shown in orange. The four isotopologues (^13^C_0–3_) of methyl acetate formed are shown.

In this study, we further evaluated the hypothesis that methyl acetate is derived from the acetylation of methanol by acetate/acetyl-CoA using labeling poplar (*Populus trichocarpa*), a fast-growing biofuel crop, simultaneously with both [1,2-^13^C]acetate and [^13^C]methanol delivered through the transpiration stream (Figure 1). This hypothesis predicts that if ^13^C-labeled methanol and acetate are delivered in equal amounts sufficient to overwhelm natural unlabeled pools in poplar branches, triply labeled [^13^C_3_]methyl acetate will be the dominant isotopologue of emitted methyl acetate. Moreover, we further evaluate the hypothesis that drought and high temperatures stimulate the acetate fermentation pathway using methyl acetate emissions as a non-invasive tracer of increased acetate production (Figure 1). We quantified methyl acetate emissions during the rapid leaf desiccation following detachment in the light (timescale of hours) and continuously during the day and night from branches of potted seedlings following the cessation of daily soil moisture additions (timescale of days). Finally, the temperature responses of methyl acetate emissions were quantified from physiologically active branches of potted saplings inside a temperature-controlled growth chamber with constant light/dark cycles and diurnally changing air temperature.

## 2. Results

### 2.1. Quantification of Gas-Phase Methyl Acetate

In order to accurately quantify methyl acetate emissions from leaves and branches, we calibrated the proton transfer reaction-mass spectrometry (PTR-MS) m/z 75 response to a gas standard prepared by dynamically diluting a primary standard (1.0 ppm methyl acetate in nitrogen). An example six-point calibration curve is shown in Figure S1, demonstrating the high sensitivity (slope: 1.1 × 10^5^ ppb ncps (normalized counts per second)_m/z 75_^−1^ = 91 cps (counts per second)_m/z 75_ ppb^−1^ at 1.0 × 10^7^ cps_m/z 21_) and linearity (R^2^ = 0.999) of the PTR-MS m/z 75 response over the wide range of gas-phase concentrations anticipated in plant enclosure experiments (0–45 ppb). The presence of methyl acetate emissions in the enclosure headspace during leaf desiccation experiments was verified using thermal desorption gas chromatography-mass spectrometry (GC-MS; Figure S2). The GC-MS data followed the same temporal trend as the PTR-MS data, but had a lower magnitude. Given the low time resolution of the GC-MS system (1 sample every ~45 min), PTR-MS was used to quantify total methyl acetate emissions by integrating the emissions over time as well as real-time emissions during the ^13^C-labeling, temperature responses, and drought experiments. However, given that empty plant enclosures showed a very low to negligible background of methyl acetate on both analytical systems, the results demonstrate that the molecular weight ion (PTR-MS: 75, GC-MS: 74) can be used to quantify gas-phase methyl acetate concentrations originating from foliar emissions into dynamic gas exchange enclosures and determine ^13^C-incorporation into each of the three carbon atoms.

### 2.2. Monitoring Methyl Acetate Formation Using ^13^C-labeling

**Figure 2:**
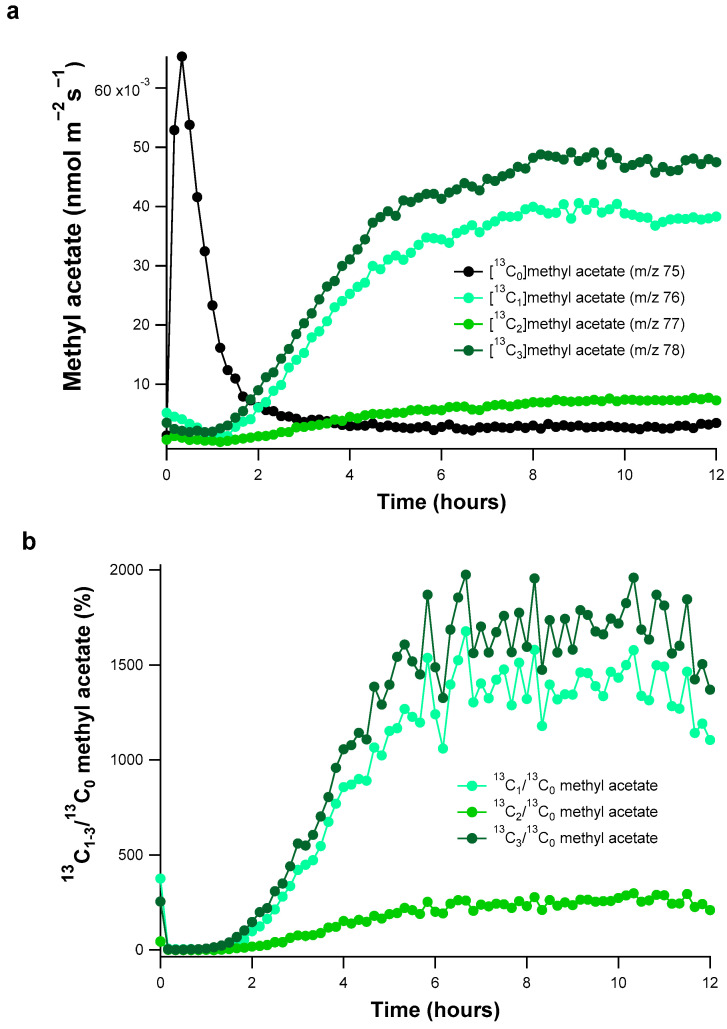
Real-time branch emissions in the light of methyl acetate isotopologues (nmol m^−2^ s^−1^) with 0–3 ^13^C-atoms and their stable carbon isotope ratios (^13^C_1–3_/^13^C_0_, %) during the delivery of a 10 mM [^13^C]methanol and [^13^C_2_]acetate solution to a detached poplar branch via the transpiration stream. (**a**) Emissions of methyl acetate isotopologues monitored using proton transfer reaction-mass spectrometry (PTR-MS) over the 12-h labeling period (10 min averages). Emissions of four isotopologues of methyl acetate were analyzed including unlabeled (^13^C_0_), singly labeled (^13^C_1_), doubly labeled (^13^C_2_), and triply labeled (^13^C_3_). (**b**) The ^13^C_1–3_/^13^C_0_ methyl acetate carbon isotope ratios (%) calculated from the real-time emission data shown in part a.

The results of the ^13^C-labeling studies demonstrate that the PTR-MS analysis of headspace air is able to resolve the emission rates of four distinct methyl acetate isotopologues from poplar branches in real-time (Figure 2 and Figure S3). Given that methyl acetate is hypothesized to derive from the acetylation of methanol via acetate/acetyl-CoA in a 1/1 ratio, we delivered a solution containing equal concentrations of [^13^C]methanol and [^13^C_2_]acetate (10 mM) to detached poplar branches via the transpiration stream in order to evaluate this hypothesis. Branch emissions of natural ^12^C-methyl acetate, referred to here as [^13^C_0_]methyl acetate, and labeled [^13^C_1–3_]methyl acetate, showed strong responses to the delivery of [^13^C]methanol and [^13^C_2_]acetate to the plant transpiration stream. 

Upon placing the branch in the enclosure, the natural emissions of [^13^C_0_]methyl acetate declined to very low levels (0.0028 ± 0.0003 nmol m^−2^ s^−1^ after 6 h) and were replaced after several hours with triply ^13^C-labeled [^13^C_3_]methyl acetate as the dominant methyl acetate isotopologue (Figure 2a). In addition, while slightly lower in magnitude than the triply ^13^C-labeled isotopologue, emissions of singly ^13^C-labeled [^13^C_1_]methyl acetate were also strongly stimulated. Both [^13^C_3_]methyl acetate and [^13^C_1_]methyl acetate emissions reached a steady state between 6–13 h after placing the branch in the ^13^C-solution, and slightly declined in magnitude over the last hour of the experiment (11–12 h). These emission trends resulted in the stable carbon isotope ratios of singly labeled (^13^C_1_/^13^C_0_) and triply labeled (^13^C_3_/^13^C_0_) methyl acetate reaching values up to 1673% and 1982%, respectively (Figure 2b). While the doubly labeled [^13^C_2_]methyl acetate emissions were also stimulated, important differences can be noted in both its emission magnitude and trend relative to [^13^C_1_] and [^13^C_3_]methyl acetate (Figure 2). In contrast to [^13^C_1_] and [^13^C_3_]methyl acetate which reached a steady state after 6 h, emissions of doubly labeled [^13^C_2_]methyl acetate slowly increased throughout the experiment, but remained substantially lower in magnitude than [^13^C_1_] and [^13^C_3_]methyl acetate reaching maximum ^13^C_2_/^13^C_0_ values of 290% (Figure 2b).

### 2.3. Methyl Acetate Emissions during Desiccation of Detached Leaves

**Figure 3:**
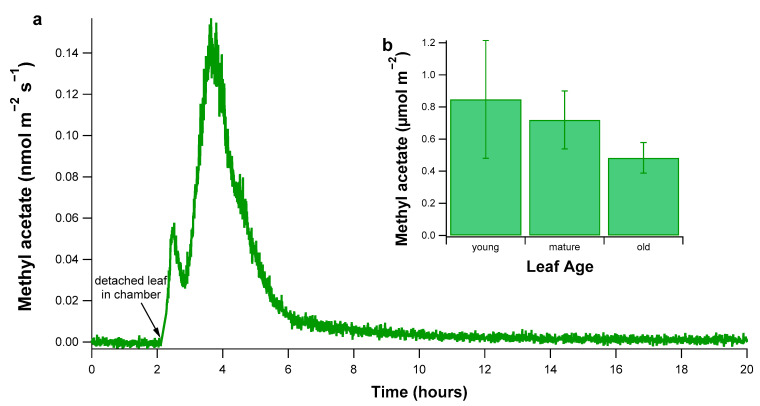
Methyl acetate emissions from detached poplar leaves during desiccation. (**a**) Methyl acetate emissions quantified over time following the introduction of the detached poplar leaves (2) inside the illuminated glass chamber with dry hydrocarbon-free air flowing through. Note the time when the detached leaves were introduced into the chamber is depicted on the plot with an arrow. (**b**) Summary of total methyl acetate emitted from detached poplar leaves for young (n = 9), mature (n = 7), and old (n = 6) leaf age classes.

Detached leaf assays were performed to simulate a rapid desiccation of illuminated poplar leaves during an extreme drought and to quantify the resulting total methyl acetate emissions from three developmental classes of poplar leaves (young, mature, and old). For each leaf class, a similar temporal pattern of methyl acetate emissions was observed (see example in Figure 3a). Upon placing the detached leaves in the illuminated glass leaf chamber, a large release of methyl acetate emissions was observed, lasting up to 1 h. This was followed by a much larger emission peak associated with the release of water vapor during the desiccation process that lasted for 3–4 h. Methyl acetate emissions returned to background levels with emissions undetectable within 10 h of placing the leaves in the chamber. When the total amount of methyl acetate emitted per m^2^ of leaf was calculated for each age class, methyl acetate emissions were found to decrease with the leaf age, with emissions from old leaves decreasing by 44% relative to emissions from young leaves (Figure 3b). Total methyl acetate emissions for each age class were as follows: Young leaves (0.85 ± 0.37 µmol m^−2^), mature leaves (0.72 ± 0.18 µmol m^−2^), and old leaves (0.48 ± 0.10 µmol m^−2^). The difference between mature and old leaves was statistically significant (*p* = 0.040, ANOVA and Tukey post-hoc analysis).

### 2.4. Intact-Branch Methyl Acetate Emissions under Increasing Temperature and Drought

The previously discussed experiments considered detached poplar branches exposed to a solution containing ^13^C-labeled methanol and acetate (Figure 2), or detached leaves undergoing rapid desiccation in the light, which typically was complete within 3–4 h (Figure 3). Next, continuous intact branch gas exchange studies, lasting up to 11 days, were conducted in order to investigate the environmental controls (temperature and soil moisture) over natural methyl acetate emissions from physiologically active poplar saplings. For these experiments, individual trees were placed inside an environmentally controlled growth chamber with constant light intensity during the day and a diurnal program of continuously changing air temperature. The trees were kept well-watered throughout the temperature experiments and were well-watered prior to the drought experiment. It should be noted that the purified air delivered to the branch enclosure contained ambient CO_2_ and H_2_O levels of the laboratory room, which varied with laboratory ventilation cycles. Nonetheless, when the intact branch was placed in the enclosure, photosynthesis and transpiration were evidenced by a significant drawdown of the enclosure CO_2_ and a large increase in the enclosure H_2_O concentrations, respectively.

**Figure 4:**
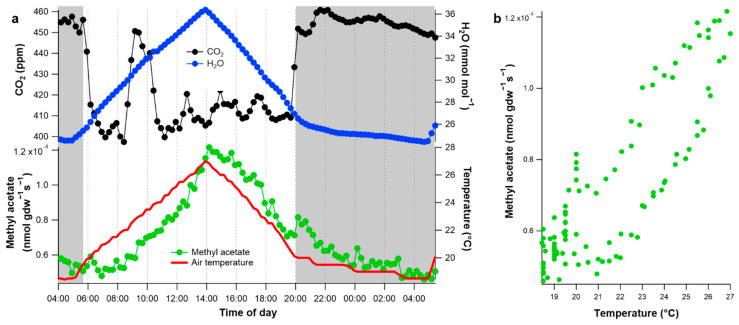
Diurnal patterns of methyl acetate emissions from poplar under increasing temperatures. Methyl acetate emissions were monitored over a 24-h period from a physiologically active branch on a potted poplar tree within a temperature-controlled growth chamber with a constant light intensity during the day, but a diurnal increase in air temperature. (**a**) Time series of headspace CO_2_ and H_2_O concentrations inside the dynamic branch enclosure together with methyl acetate emissions and air temperature. The greyed areas represent the night period when the growth chamber light was off. (**b**) Branch emissions of methyl acetate plotted versus air temperature.

Intact branch methyl acetate emissions were low, reaching a maximum of 1.2 × 10^−4^ nmol gdw^−1^ s^−1^ (7.7 × 10^−3^ nmol m^−2^ s^−1^) in the early afternoon (Figure 4a). Thus, natural diurnal emissions of methyl acetate were 10–20 times lower than maximum methyl acetate emission rates measured during the detached leaf desiccation experiments (Figure 3). Methyl acetate followed a clear diurnal pattern along with air temperature, but did not track the branch enclosure temperature as tightly as water vapor. Transpiration is tightly linked with temperature due to its strong impact over vapor pressure deficit, the driver of leaf transpiration. In contrast, methyl acetate emissions seemed to slightly lag behind changes in air temperature, but nonetheless showed a clear positive correlation with air temperature (Figure 4b). Although small, methyl acetate was also observed to be emitted at night, as seen by the increase compared to background levels, this is likely due to poplar not closing the stomata fully during the night.

**Figure 5:**
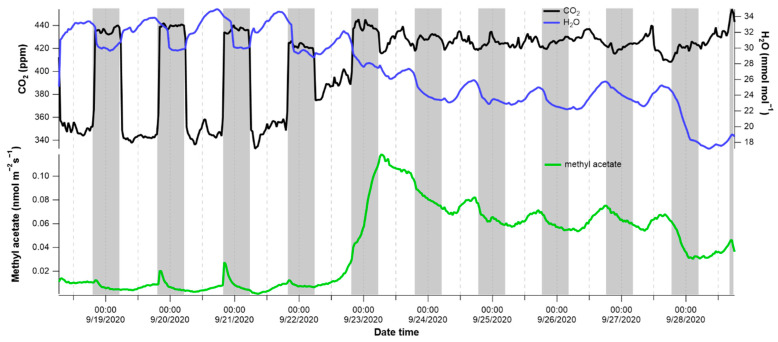
Methyl acetate emissions during drought. Branch methyl acetate emissions together with headspace concentrations of CO_2_ and H_2_O monitored continuously over 10 days with soil water additions withheld for the entire experiment. The greyed areas represent the night period where the LED-grow light was off.

In order to assess the effect of drought on methyl acetate emissions together with transpiration and photosynthesis activity, we monitored the gas exchange continuously for up to 10 days after withholding water from previously well-watered poplar saplings. Plants that were transferred from the greenhouse to the laboratory just following a watering event took up to 3 days to show drought effects on the branch gas exchange (Figure 5 and Supplementary Figures S4 and S5). In contrast, the plant that was collected just prior to the daily morning watering in the greenhouse showed drought effects within the same day (Figure S6). An example drought experiment is shown in Figure 5, where a strong draw down in the chamber CO_2_ concentrations (440 → 340 ppm) during photosynthesis in the light were observed for the first 3 days following the cessation of watering. This corresponded to increased transpiration rates during the day as evidenced by the higher water vapor concentrations inside the enclosure. It should be noted that while water vapor concentrations decreased substantially during the night, there was considerable transpiration still occurring as evidenced by the relatively higher water vapor concentrations at night (29–30 mmol mol^−1^) as compared to the air entering the chamber (16–18 mmol mol^−1^) (Figure 5). This is consistent with previous studies showing that poplar does not fully close its stomata in the dark allowing transpiration to continue at night. Thus, during the first 4 days of the experiment, the poplar sapling showed no signs of water deficit stress, with a strong uptake of CO_2_ and release of H_2_O during daylight hours, indicating a highly physiologically active plant. During this non-stressed period, branch emissions of methyl acetate emissions were low (<0.02 nmol m^−2^ s^−1^), typically reaching its highest daytime emission rate at the end of the light period, emissions were also low in a control non-drought stressed tree (Figure S7). As temperature inside the branch chamber was not controlled during the drought experiments, this is likely due to a slight gradual warming inside the branch enclosure due to the light absorption. Isoprene emissions, which are also known to be strongly stimulated by temperature in the light [21], showed the same pattern (Figure S8). Moreover, prior to drought-induced stress during the first 3–4 days, clear emission bursts of methyl acetate (up to 0.03 nmol m^−2^ s^−1^) were often stimulated following the sudden switching off of the light at the start of the dark period. In contrast, these emission bursts following the light-dark transition were eliminated once the drought effects set in (e.g., days 6–11). Drought stress was evident from day 5, with a marked reduction in CO_2_ uptake and transpiration was observed through a reduced uptake of enclosure CO_2_ and H_2_O buildup during the day. This was associated with a large increase in methyl acetate emissions throughout the night and peaking the following day (up to 0.12 nmol m^−2^ s^−1^). These enhanced emissions remained high throughout the rest of the experiment when no clear net uptake in CO_2_ could be observed and H_2_O vapor concentrations continued to decline until they reached the value of the incoming air (16–18 mmol mol^−1^) on day 11. Although the timing and magnitude of the drought-induced methyl acetate emissions varied from replicate drought experiments on potted poplar saplings, (Supplementary Figures S4–S6), similar patterns were observed with the onset of drought response associated with the suppressed uptake of CO_2_ and release of H_2_O together with a stimulation of methyl acetate emissions. The initiation of these increased drought-induced methyl acetate emissions was observed both in the day and night.

## 3. Discussion

It has been hypothesized that the acetylation of cell wall-derived methanol, the simplest primary alcohol highly abundant in C_3_ and C_4_ plants, yields the volatile product methyl acetate. However, evidence for this is lacking, and plant methyl acetate emission observations are scarce, with the influence of developmental and environmental variables unknown. In this study, we provide the first confirmation that foliar emissions of methyl acetate derive from the acetylation of methanol by delivering a [^13^C]methanol and [^13^C_2_]acetate solution in equal concentrations (10 mM) to the transpiration stream of detached poplar branches. Emission of the triply labeled [^13^C_3_]methyl acetate as the dominant isotopologue from the poplar branch during the ^13^C-labeling is consistent with a mechanism by which [^13^C]methanol is acetylated to [^13^C_3_]methyl acetate by [^13^C_2_]acetyl CoA, catalyzed using one or more alcohol *O-*acetyltransferase enzymes [22]. For example, characterization of the alcohol acyltransferase AT9 from kiwifruit (*Actinidia* spp.) demonstrated acetyl-CoA:alcohol *O*-acyltransferase activity with a strong preference for short chain alcohol substrates [23]. This mechanism requires the delivered [^13^C_2_]acetate to be first activated to [^13^C_2_]acetyl-CoA by acetyl-CoA synthetase. A key component of acetate metabolism in plants responsible for the conversion of acetate fermentation products (acetaldehyde, ethanol, and acetate) to acetyl-CoA derived metabolites [24]. Recently, studies with *Arabidopsis* double mutants of ACETYL-COA SYNTHETASE (ACS) in plastids and ACETATE NON-UTILIZING1 (ACN1) in peroxisomes (which enables the incorporation of acetate into organic acids and amino acids) showed severe morphological and metabolic phenotypes including delayed growth and sterility associated with hyperaccumulation of cellular acetate and decreased accumulation of acetyl-CoA-derived intermediates of central metabolism [25].

It is interesting to note that strong emissions of singly labeled [^13^C_1_]methyl acetate were also observed from the poplar branches during the ^13^C-labeling. Although PTR-MS cannot distinguish between the one methoxy and two acetyl carbon atoms of methyl acetate, the results suggest that this methyl acetate isotopologue was derived from the acetylation of the delivered [^13^C]methanol with natural unlabeled acetyl-CoA pools within the poplar branch. In addition, the clear but smaller emissions of doubly labeled [^13^C_2_]methyl acetate, likely derived from the acetylation of natural cell wall-linked methanol with [^13^C_2_]acetyl CoA produced from the supplied [^13^C_2_]acetate. Thus, the delivered [^13^C]methanol overwhelmed the natural unlabeled methanol pool(s) with up to 91% of total methyl acetate emissions containing a ^13^C-methoxy carbon ([^13^C_1_]methyl acetate + [^13^C_3_]methyl acetate). In contrast, the ^13^C_2_-acetyl moiety of methyl acetate ([^13^C_2_]methyl acetate + [^13^C_3_]methyl acetate) resulted in a maximum of 62% of the total methyl acetate emissions. These results are consistent with methanol as the main substrate for the methoxy moiety and acetate/acetyl-CoA, a potentially dominant source of *O*-acetyl moieties in methyl acetate emissions [1].

Real-time branch gas exchange observations in poplar saplings before and during the experimental drought revealed a loss of net CO_2_ assimilation and decreases in transpiration. In contrast, methyl acetate emissions were stimulated by drought and coincided with altered photosynthesis and water use. Taken together, our labeling results and previous work [1,26], show that methyl acetate emissions derive from the intersection of cell wall derived methanol, C_1_ metabolism, and C_2_ acetate fermentation. The acetate biosynthetic pathway consists of the decarboxylation of pyruvate producing acetaldehyde which is subsequently oxidized to acetic acid and this pathway was upregulated during drought stress [15]. We propose that the observed increase in methyl acetate emissions was due to the activation of the acetate fermentation pathway, resulting in increased acetate that was converted to acetyl CoA and then methylated, producing methyl acetate. This pathway has been implicated in the plant stress response [27,28,29]. Activation of the acetate fermentation pathway has been shown to be important for drought tolerance in *Arabidopsis* [15,16].

By directly measuring methyl acetate emission patterns, our study supports previous findings of a drought stimulation of acetate-linked volatile production and emissions from plants (volatile acetate esters). Methyl acetate emissions from poplar branches increased with temperature, as has been reported for other VOCs including methanol, monoterpenes, and isoprene [30,31]. The phenological pattern of decreasing methyl acetate emissions from older leaves is reflected in the phenological dependence of methanol and acetic acid emissions [13]. In other studies, the terpene acetylation content of *Cupressus sempervirens* leaves during drought showed that while mono- and sesquiterpenes and terpenols were nearly completely metabolized, α-terpinyl acetate accumulated during the first month of water stress, reaching double its initial content [32]. In another study, strong enhancements in leaf (Z)-3-hexenyl acetate emissions, corresponding with the loss of net photosynthesis, were observed in a tropical tree in the central Amazon following leaf detachment in the canopy [33] and (E)-2-hexenyl acetate emissions were drought stimulated in potted apple trees (*Malus domestica* [L.] Borkh. ev. Delicious) [34].

## 4. Conclusions

It is now recognized that an activation of acetate fermentation in plants is a key evolutionarily conserved drought survival strategy that coordinates changes in transcription, cellular metabolism, and hormone signaling and the associated physiological changes including altered plant carbon cycling and water use. In *Arabidopsis*, the whole regulatory network depends on histone acetylation, in which acetate fermentation primes the jasmonate signaling pathway for enhanced drought tolerance. Given the increased frequency, intensity, and duration of widespread climate-linked drought and high heat anomalies documented globally, characterizing acetate fermentation and gas exchange responses to drought in plants and ecosystems across diurnal, seasonal, and interannual time scales is of critical importance. Although the amount of acetate produced in plant tissues has been directly linked with increased drought survival, studies reporting the dynamics of plant acetate fermentation responses to drought remain scarce. Destructive measurements are required to evaluate acetate-linked drought responses, limiting the biological variables, and temporal and spatial scales that can be studied for drought responses. Here, we used ^13^C-labeling of poplar branches to demonstrate acetate as a major source of acetyl-CoA pool(s) utilized during the methyl acetate biosynthesis. The labeling pattern of methyl acetate branch emissions during the delivery of a [^13^C]methanol and [^13^C_2_]acetate solution to the transpiration stream is consistent with the acetyl-CoA:alcohol *O*-acyltransferase activity of alcohol *O-*acetyltransferase enzymes, with a preference for short chain alcohol substrates such as methanol.

The strong stimulation of branch methyl acetate emissions was coincident with the loss of net carbon assimilation and reduced transpiration and continued at elevated levels for days. The results suggest that the activation of the acetate fermentation pathway coincides with drought-induced stomatal closure, and the suppression of net photosynthesis and transpiration. Thus, methyl acetate emissions may represent a novel non-invasive tracer of the activation of acetate-mediated survival responses to environmental extremes (e.g., drought and heat) and the corresponding changes to carbon cycling and water use in poplar. Strong increases in canopy methyl acetate emissions in poplar plantations could be interpreted as an early “warning” signal of ecosystem drought stress response including the activation of acetate fermentation and reductions in net primary productivity and transpiration. Future studies quantifying acetate-mediated drought responses in poplar plantations could therefore include methyl acetate emissions with more common gas exchange measurements (e.g., CO_2_ and H_2_O) across biological (e.g., species), spatial (leaf to ecosystem), and temporal (diurnal to annual) scales. It would also be valuable to compare methyl acetate emissions from a range of species across ecosystems to ascertain how conserved this phenomenon is. To further explore the mechanistic links between methyl acetate emissions, acetate fermentation, and carbon cycling and water use, leaf datasets on wild-type and transgenic poplar with modified acetate metabolism would be of high value. This includes leaf data sets linking methyl acetate emissions with plant hydraulics, cell wall physicochemical properties, cellular metabolism (photosynthesis, respiration, fermentation), jasmonate hormone signaling, and transcription.

## 5. Materials and Methods

### 5.1. Plant Material 

Twenty poplar (black cottonwood: *Populus trichocarpa*) saplings were obtained from a commercial supplier (Plants of the Wild, Tekoa, WA, USA). The trees were transferred into #2 pots (6.59 L) with Supersoil planting media (Scotts Co., Marysville, OH, USA) and maintained in the UC Berkeley Oxford Tract greenhouse under natural lighting supplemented with LED lighting (6:00–20:00 light period; Lumigrow 325 Pro, Emeryville, CA, USA). Individual plants were transferred to environmentally controlled growth chambers (Percival Intellus Control System, Perry, IA, USA) during active experiments maintained at 27.5 °C daytime temperature (5:45 a.m.–20:00 p.m.; 30% light) and 23 °C nighttime temperature (20:00 p.m. to 5:45 a.m.). Leaf age was determined as in [35], with young leaves defined as not fully expanded and light green in color, mature leaves fully expanded and dark green, and old leaves showed signs of brown senescence on their edges.

### 5.2. Online PTR-MS (Proton Transfer Reaction Mass Spectrometry)

Methyl acetate gas-phase concentrations exiting the dynamic leaf and branch chambers were quantified in real-time using a high sensitivity quadrupole proton transfer reaction mass spectrometer (PTR-MS, Ionicon, Innsbruck Austria, with a QMZ 422 quadrupole, Balzers, Switzerland), as in [13]. The PTR-MS was operated with a drift tube voltage of 600 V and pressure of 1.9 mb. The following mass to charge ratios (m/z) were sequentially monitored during each PTR-MS measurement cycle: (1) m/z 32 (O_2_^+^) and 37 (H_2_O-H_3_O+) with a 10 ms dwell time, (2) m/z 21 (H_3_^18^O^+^) with a 50 ms dwell time, (3) m/z 25 (dark counts), (4) m/z 75 ([^13^C_0_]methyl acetate), m/z 76 ([^13^C_1_]methyl acetate), m/z 77 ([^13^C_2_]methyl acetate), and m/z 78 ([^13^C_3_]methyl acetate) with a 5 s dwell time each. A linear calibration curve of a methyl acetate gas standard (Restek Corporation, Bellefonte, PA, USA) was used to quantify the concentration of methyl acetate in the samples. Calibration response curves were generated for m/z 75 ([^13^C_0_]methyl acetate) for 0.0, 9.4, 19, 28, 36, and 45 ppb of the gas primary standard. During the ^13^C-labeling experiments (Section 5.3), the same response factor for [^13^C_0_]methyl acetate was also used for [^13^C_1–3_]methyl acetate. Leaf and branch emission rates of [^13^C_0–3_]methyl acetate were determined using the flow rate through the chamber, the concentration difference with and without vegetation inside the chamber, and total leaf area or dry weight in the chamber.

Methyl acetate emissions during leaf desiccation experiments were verified using thermal desorption gas chromatography-mass spectrometry [31]. Samples were passed through a Korixr at −20 °C to remove water, then methyl acetate was pre-concentrated onto a cold trap (Air Toxics, Markes International, Bridgend, UK) at −30 °C. The sample flow path was kept at 150 °C. The methyl acetate sample was then injected onto a capillary column (Rtx-VMS, 60 m × 0.25 mm × 1.4 μm) interfaced with a gas chromatograph (7890B, Agilent Technologies, Santa Clara, CA, USA) with a high efficiency source electron impact quadrupole mass spectrometer (5977B HES MSD, Agilent Technologies, Santa Clara, CA, USA). While the sample was injected onto the column, the cold trap was heated to 280 °C and held for 3 min with back-flushing of the carrier gas (6.5 mL min^−1^) of which 1.5 mL min^−1^ was directed onto the column. The temperature of the column was initially held at 40 °C for 1.5 min, then ramped up to 170 °C at a rate of 15 °C min^−1^. Following the run, the temperature was held at 230 °C for 1.5 min. The mass spectrometer was configured for trace analysis (SIM Mode and 10× detector gain factor) with 50 ms dwell times for methyl acetate (m/z 74, 43, 59). Verification of methyl acetate in the plant enclosure air samples was based on a comparison of mass spectra and retention time (7.6 min) of samples with those of a primary methyl acetate gas standard (Restek Corporation, Bellefonte, PA, USA). 

### 5.3. [13. C_2_]acetate and [^13^C]methanol Labeling of Poplar 

During ^13^C-labeling experiments of methyl acetate branch emissions, a branch was detached from a potted poplar with the stem immediately recut while submerged in a solution of 10 mM [^13^C_2_]acetate and 10 mM [^13^C]methanol (Sigma-Aldrich, St Louis, MO, USA). The top part of the branch (nine leaves, 17.4 g fresh weight) was placed inside a dynamic 5.0 L Tedlar branch enclosure (CEL Scientific Corporation, Cerritos, CA, USA) with 2.0 L min^−1^ of hydrocarbon free air passing through and illuminated using an LED growth light (1000–1500 µmol m^−2^ s^−1^ photosynthetic photon flux density) to encourage transpiration and photosynthesis. A fraction of the air exiting the chamber (~75 mL min^−1^) was directed to the online PTR-MS system and used to monitor the emissions of methyl acetate isotopologues with 0–3 ^13^C atoms. ~30 mL of the solution was taken up by the branch over 14 h (Figure 1). The experiment was repeated twice, with the second experiment lasting 3 days and the branch taking up ~140 mL of the solution (Figure S3).

### 5.4. Detached Leaf Desiccation Methyl Acetate Emission Assay

The detached leaf assays were carried out as in [13]. Briefly, detached poplar leaves were desiccated in a 475-mL glass chamber and the emissions of methyl acetate were monitored in real-time using PTR-MS (Section 5.2). An LED light (1000–1500 µmol m^−2^ s^−1^ photosynthetic photon flux density) was positioned above the chamber to aid desiccation. Dry hydrocarbon-free air (300 mL min^−1^) was passed through the chamber, in which 75 mL min^−1^ was then sampled by PTR-MS. Background levels of methyl acetate from the empty chamber were monitored for approximately 2 h. Two leaves of the same age class were detached from a poplar tree and immediately placed in the glass chamber, where methyl acetate emissions were then quantified for 18 h. Replicate pairs of young (n = 9), mature (n = 7), and old (n = 6) leaves were analyzed.

### 5.5. Temperature and Drought Treatment of Potted Poplar Saplings 

Real-time dynamics of methyl acetate emissions together with photosynthesis and transpiration were collected from physiologically active poplar trees under constant daytime light with (1) controlled diurnal air temperature and (2) drought. To evaluate the temperature sensitivity of foliar methyl acetate emissions, individual poplar trees were placed in an environmentally controlled growth chamber with a light period (20% light intensity, 6:00 a.m.–20:00 p.m.) with air temperature linearly changing between a minimum at 5:00 a.m. (20 °C) to a maximum at 14:00 p.m. (30 °C). A potted polar sapling was transported from the greenhouse to the laboratory and placed in the growth chamber, with the top part of one branch (3.8 g dry weight (gdw) leaves) placed inside a dynamic 5.0 L Tedlar branch enclosure with 2.0 L min^−1^ of hydrocarbon free air passing through. A small fraction of the air exiting the chamber was diverted to a PTR-MS (75 mL min^−1^) for methyl acetate concentration determination and an infrared gas analyzer (Li7000, Licor Biosciences, Lincoln, NE, USA) for CO_2_ and H_2_O concentration measurements. Air temperature inside the branch enclosure was recorded using a type-T thermocouple with the sensor shaded from direct light using white Teflon tape. Following the collection of background data with no branch in the enclosure, diurnal gas exchange data were collected. This experiment was repeated on three different poplar saplings. 

A similar experimental setup was used for poplar gas-exchange responses to drought with the exception that the experiment was conducted directly in the laboratory without the use of the growth chamber. Potted poplar saplings transferred to the laboratory were placed under an LED grow light such that the top of the branch to be studied for gas exchange was exposed to a photosynthetic photon flux density of 800–1200 µmol m^−2^ s^−1^. Daily soil moisture additions in the morning and evening, which all plants received in the greenhouse, were withheld and the branch gas exchange was monitored continuously as described for the temperature experiments. The gas exchange was monitored continuously for 2–10 days for each of the four plants studied, depending on how quickly the drought impacts on the gas exchange were observed.

### 5.6. Statistical Analysis

Statistical analyses were performed in R version 4.0.0. ANOVA with the Tukey post-hoc analysis was used to determine the statistical difference between total methyl acetate emissions of different leaf age classes (young n = 9; mature n = 7; old n = 6). Statistical significance was defined as *p* < 0.05.

## Data Availability

Data presented in this article, including raw methyl acetate emissions data obtained from PTR-MS, and CO_2_ and H_2_O concentrations obtained from Licor7000 are accessible free of charge through Mendeley Data (doi:10.17632/rzpn9bck6w.1).

## Supplementary Materials

The following are available online at https://www.mdpi.com/2223-7747/10/2/411/s1. Figure S1: Example PTR-MS response curve to a six-point calibration (0–47 ppb); Figure S2: Example of methyl acetate emissions monitored by coupled PTR-MS and GC-MS systems; Figure S3: [^13^C_0–3_] methyl acetate emissions from a detached poplar branch during the delivery of a 10 mM [^13^C]methanol and [^13^C_2_]acetate solution; Figures S4–S6: Branch methyl acetate emissions together with headspace concentrations of CO_2_ and H_2_O following the cessation of daily soil additions; Figure S7: Branch methyl acetate emissions from a drought stressed treecompared to a control non-stressed tree.; Figure S8: Diurnal isoprene emissions over a 24-h period from a physiologically active branch on a potted poplar tree during diurnal temperature response experiments.
