# Supplementary material for: Are Methanol-Derived Foliar Methyl Acetate Emissions a Tracer of Acetate-Mediated Drought Survival in Plants?
Source: Plants (Basel). 2021 Feb 23;10(2):411. doi: 10.3390/plants10020411 (PMC7927132; doi:10.3390/plants10020411)
Supplement: Supplementary file 1 [file plants-10-00411-s001.pdf]

plants

Article

Are Methanol-Derived Foliar Methyl Acetate Emissions a Tracer of Acetate-Mediated Drought Survival in Plants?

Rebecca A Dewhirst [1,*], Joseph Lei[1], Cassandra A Afseth [1,2], Cristina Castanha [1], Christina M Wistrom [3], Jenny C Mortimer [4,5], and Kolby J Jardine [1]

1 Climate and Ecosystem Sciences Division, Lawrence Berkeley National Laboratory, Berkeley, CA, USA
2 School of Integrative Biology, University of Illinois, Urbana-Champaign, IL, USA
3 College of Natural Resources, University of California, Berkeley, CA, USA
4 Environmental Genomics and Systems Biology, Biosciences Division, Lawrence Berkeley National Laboratory, Berkeley, CA, USA
5 School of Agriculture, Food and Wine, Waite Research Institute, University of Adelaide, Glen Osmond, SA, Australia

* Correspondence: radewhirst@lbl.gov

Keywords: biogenic, volatile organic compound, acetylation, methanol, acetate fermentation

Supplementary Material Content

- **Figure S1**: Example PTR-MS response curve from a 6 point calibration (0-47 ppb) with a methyl acetate primary gas standard
- **Figure S2**. Example PTR-MS and GC-MS measurements of methyl acetate emissions from detached poplar leaves inside an illuminated glass chamber
- **Figure S3**: Replicate [$^{13}C_{0-3}$] methyl acetate emissions from a detached poplar branch during the delivery of a 10 mM [^{13}C]methanol and [$^{13}C_2$]acetate solution
- **Figure S4-S6:** Branch methyl acetate emissions together with headspace concentrations of CO_2 and H_2O following the cessation of daily soil water additions
- **Figure S7:** Branch methyl acetate emissions from a drought stressed tree compared to a control non-stressed tree
- **Figure S8:** Example of diurnal isoprene emissions and temperature over a 24-hour period from a physiologically active branch on a potted poplar tree during diurnal temperature response experiments.

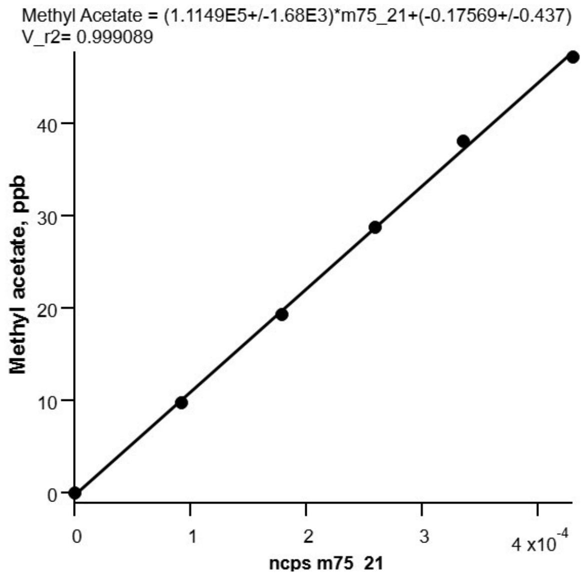

Figure S1. *Example PTR-MS response curve from a 6-point calibration (0-47 ppb) with a methyl acetate primary gas standard (Restek, USA).*

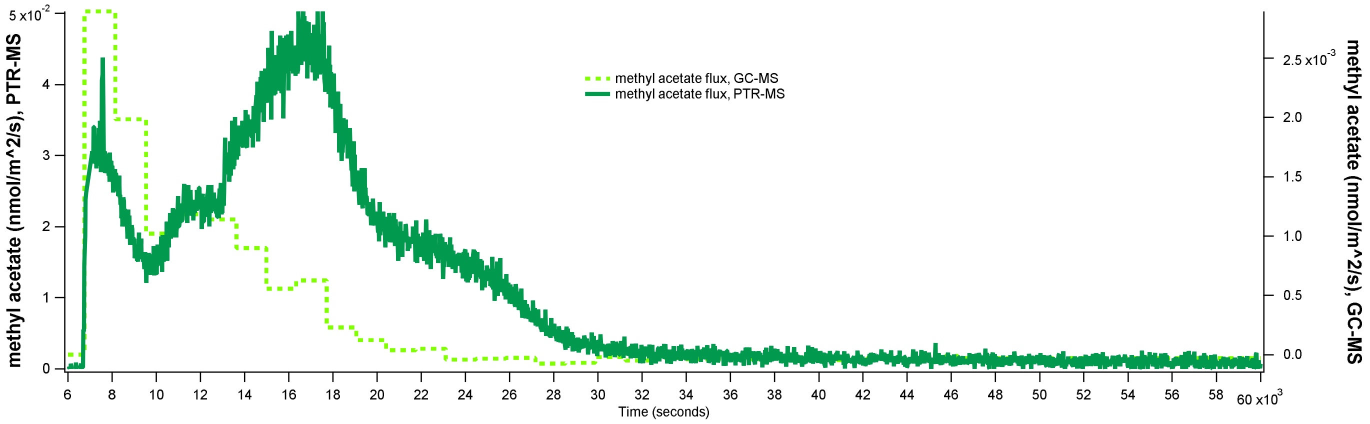

Figure S2. *Example PTR-MS and GC-MS measurements of methyl acetate emissions from detached poplar leaves inside an illuminated glass chamber with dry hydrocarbon-free air flowing through. Note that while both techniques detected similar temporal pattern in methyl acetate emissions, the GC-MS underestimated emission rates relative to PTR-MS.*

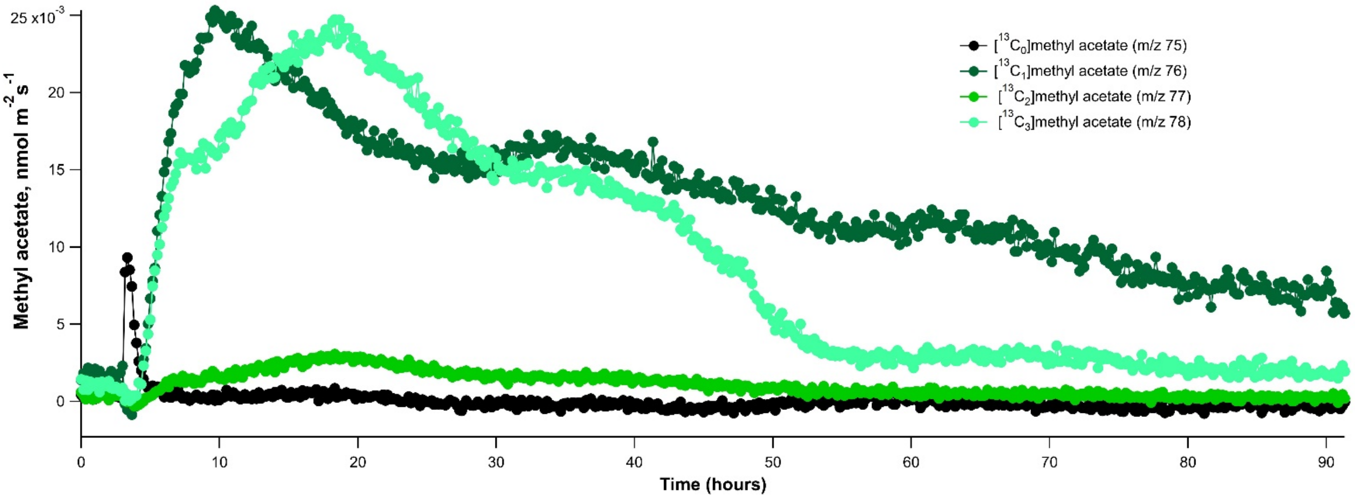

Figure S3: Real-time branch emissions of methyl acetate (nmol m^{-2} s^{-1}) during the delivery of a 10 mM [^{13}C]methanol and [$^{13}C_2$]acetate solution to a detached poplar branch via the transpiration stream. Emissions of methyl acetate isotopologues monitored using PTR-MS over the 90-hour labeling period (10 min averages). Emissions of four isotopologues of methyl acetate were analyzed including unlabeled ($^{13}C_0$), singly labeled ($^{13}C_1$), doubly labeled ($^{13}C_2$) and triply labeled ($^{13}C_3$).

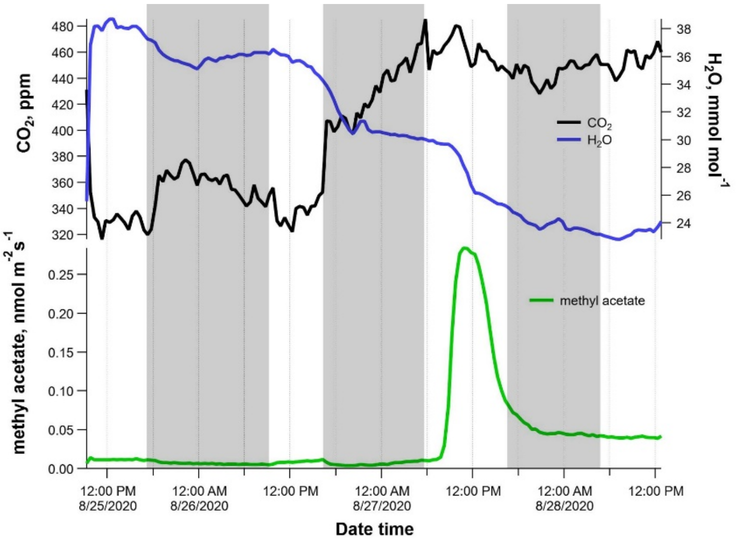

Figure S4: *Branch methyl acetate emissions together with headspace concentrations of CO_2 and H_2O monitored continuously over 3.5 days from a recently watered/hydrated potted poplar sapling with soil water additions withheld for the entire experiment. The greyed areas represent the night period where the LED-grow light was off.*

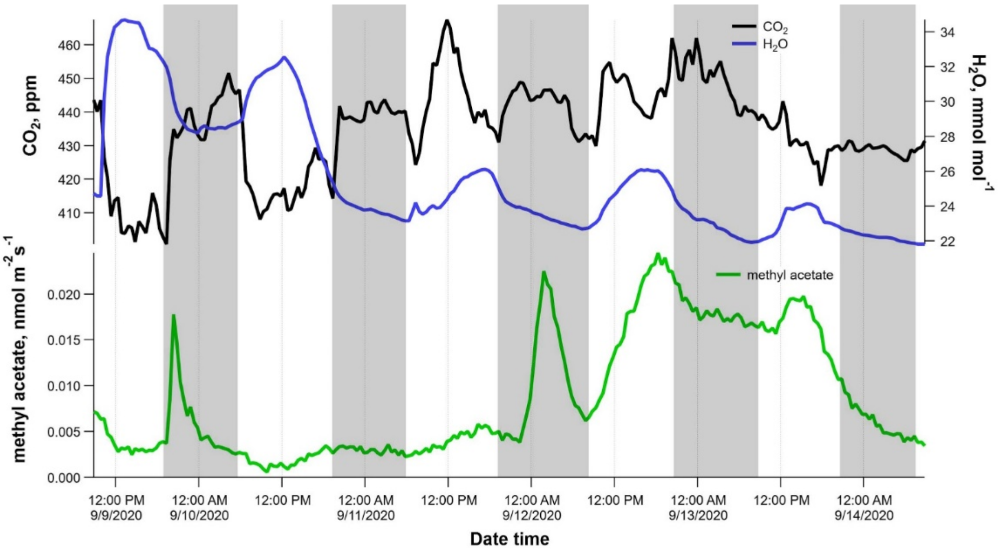

Figure S5: *Branch methyl acetate emissions together with headspace concentrations of CO_2 and H_2O monitored continuously over 5 days from a recently watered/hydrated potted poplar sapling with soil water additions withheld for the entire experiment. The greyed areas represent the night period where the LED-grow light was off.*

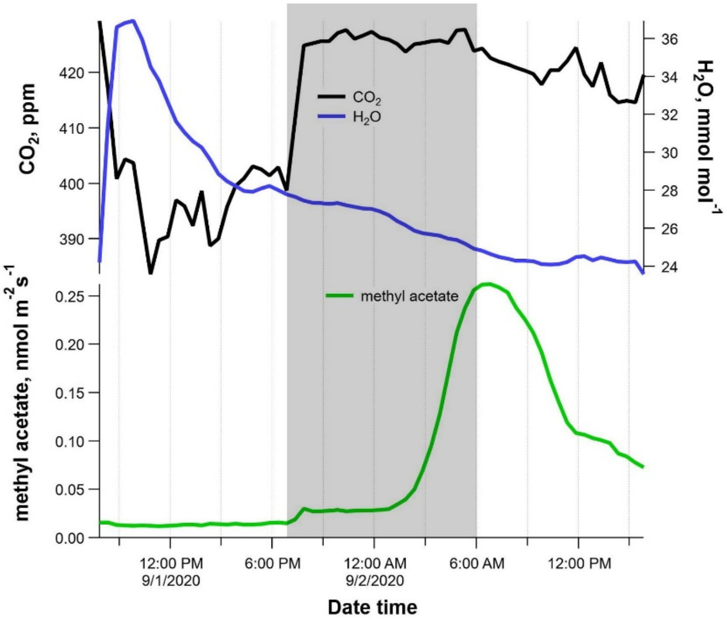

Figure S6: *Branch methyl acetate emissions together with headspace concentrations of CO_2 and H_2O monitored continuously over 1.5 days from a potted poplar sapling collected from the greenhouse prior to the morning watering and with soil water additions withheld for the entire experiment. The greyed areas represent the night period where the LED-grow light was off.*

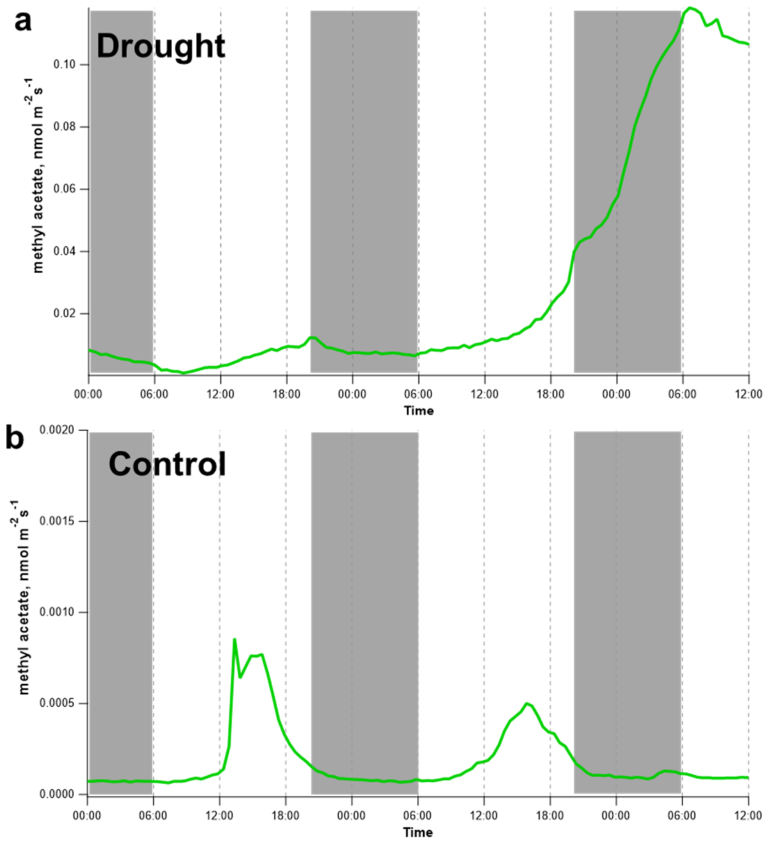

Figure S7: *Branch methyl acetate emissions from a drought stressed tree (a) compared to a control non-stressed tree (b). Note the difference between the scales, with the drought tree peaking at 0.12 nmol m^{-2} s^{-1} and the control tree peaking at 0.0008 nmol m^{-2} s^{-1}. The greyed areas represent the night period where the LED-grow light was off.*

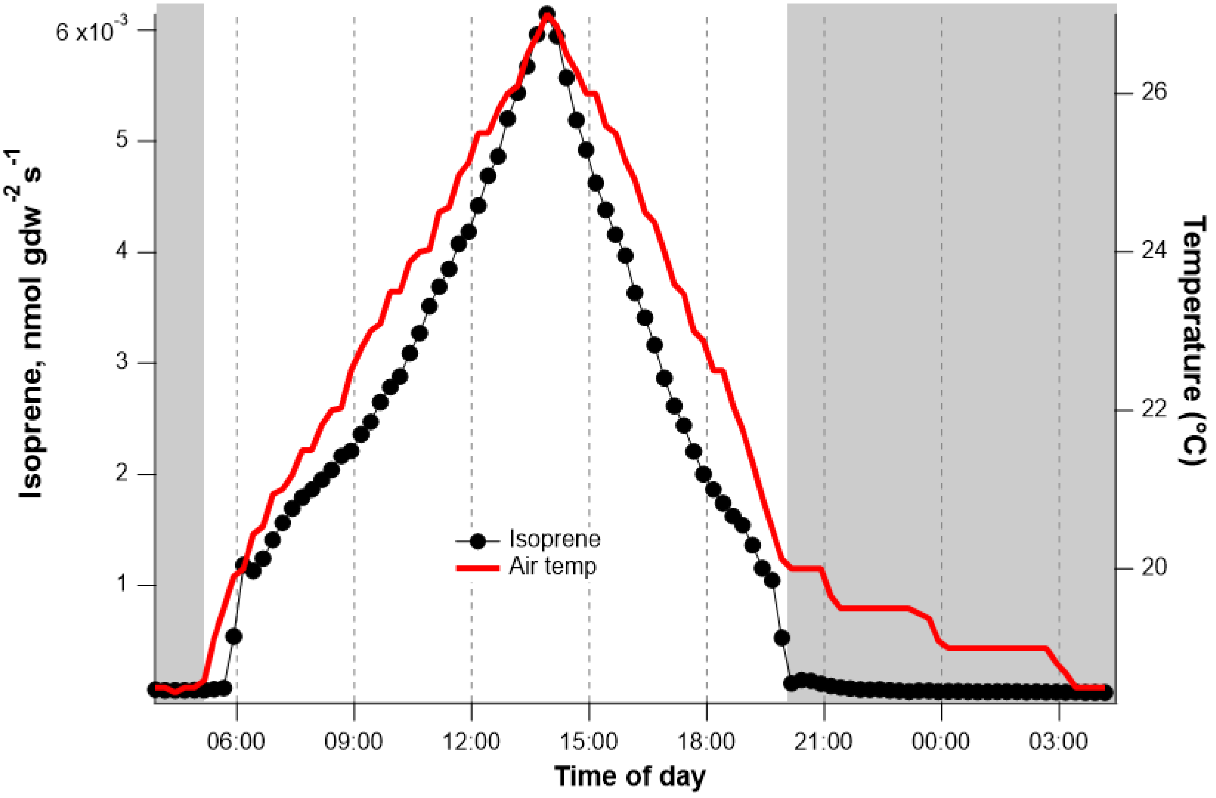

Figure S8: *Diurnal isoprene emissions over a 24-hour period from a physiologically active branch on a potted poplar tree within a temperature-controlled growth chamber with a constant light intensity during the day, but a diurnal increase in air temperature. The greyed areas represent the night period when the grow chamber light was off.*
